# Establishment of a model to predict mortality after decompression craniotomy for traumatic brain injury

**DOI:** 10.1002/brb3.3492

**Published:** 2024-04-19

**Authors:** Birui Wu, Juntao Zhang, Junchen Chen, Xibo Sun, Dianhui Tan

**Affiliations:** ^1^ Department of Neurosurgery The First Affiliated Hospital of Shantou University Medical College Shantou Guangdong China; ^2^ Department of Neurosurgery Guangdong Sanjiu Brain Hospital Guangzhou Guangdong China

**Keywords:** death, decompressive craniectomy, prediction, risk factor, traumatic brain injury

## Abstract

**Background:**

The mortality rate of patients with traumatic brain injury (TBI) is still high even while undergoing decompressive craniectomy (DC), and the expensive treatment costs bring huge economic burden to the families of patients.

**Objective:**

The aim of this study was to identify preoperative indicators that influence patient outcomes and to develop a risk model for predicting patient mortality by a retrospective analysis of TBI patients undergoing DC.

**Methods:**

A total of 288 TBI patients treated with DC, admitted to the First Affiliated Hospital of Shantou University Medical School from August 2015 to April 2021, were used for univariate and multivariate logistic regression analysis to determine the risk factors for death after DC in TBI patients. We also built a risk model for the identified risk factors and conducted internal verification and model evaluation.

**Results:**

Univariate and multivariate logistic regression analysis identified four risk factors: Glasgow Coma Scale, age, activated partial thrombin time, and mean CT value of the superior sagittal sinus. These risk factors can be obtained before DC. In addition, we also developed a 3‐month mortality risk model and conducted a bootstrap 1000 resampling internal validation, with C‐indices of 0.852 and 0.845, respectively.

**Conclusions:**

We developed a risk model that has clinical significance for the early identification of patients who will still die after DC. Interestingly, we also identified a new early risk factor for TBI patients after DC, that is, preoperative mean CT value of the superior sagittal sinus (*p* < .05).

## Graphical abstract

First, we established a model using only preoperative indicators to predict the 3‐month risk of death in TBI patients receiving DC. Undoubtedly, this can better guide our clinical work, provide a reference for future TBI treatment guidelines, and reduce the incidence of spending a large amount of money but failing to save patients.

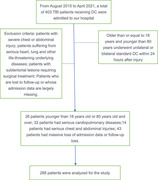

## 1 INTRODUCTION

Traumatic brain injury (TBI) is a devastating neurological disorder that is the most common cause of traumatic death worldwide (Fleminger & Ponsford, 2005; Zink, 2001). Craniocerebral injury can disturb the normal intracranial physiology (cerebrospinal fluid circulation, blood circulation, and cell metabolism), resulting in malignant intracranial pressure (ICP) as the main cause of death (Stocchetti & Maas, 2014). Studies have shown that decompressive craniectomy (DC) is an effective method to reduce ICP through partial craniotomy (Carney et al., 2017), and the effect of DC on reducing cranial pressure has been repeatedly demonstrated (Cooper et al., 2011; Jaeger et al., 2003). However, DC is also a surgical procedure associated with high postoperative morbidity and the risk of potentially serious and even life‐threatening complications (Hanko et al., 2021; Yang et al., 2008) giving rise to a large number of patients who cannot survive after DC. In addition, the high cost of surgery and postoperative monitoring treatment brings a huge economic burden to the patient's family. Therefore, whether or not to administer DC is a challenging issue that could be solved in part with the help of a prognostic scoring system and multifactor prediction model.

In recent years, machine learning methods have been gradually integrated into various fields of medicine to improve the accuracy of diagnosis and prognosis prediction. Examples include the evaluation of imaging or histopathological studies (Noyan et al., 2020) and the prediction of survival (Wongvibulsin et al., 2019) or postoperative surgical complications (Chan et al., 2006). Machine learning can be defined as the discipline that focuses on improving the ability of computers to process more data. Such methods have been used to predict the prognosis of TBI patients, such as the International Mission for Prognosis and Clinical Trials in TBI, Corticosteroid Randomization after Significant Head Injury models, and The Trauma‐Related Injury Severity Score (O'Kelly et al., 2009; Vale et al., 1997; Varelas et al., 2006). Unfortunately, few studies have modeled the prognosis of TBI patients treated with DC. Hanko et al. (2021) established a random forest prediction model to predict the 6‐month postoperative mortality in this subgroup (AUC = .811). Although this was a prospective study, the sample size was small, and there were inherent selection bias problems, so the reliability of the model still needs to be further evaluated. To this end, we sought to explore predictors of death after DC, in TBI patients, that could be obtained at admission. Based on these predictors, we developed a simple and practical death prediction model. This model can predict the risk of postoperative death before patients undergo DC.

## 2 METHODS

### 2.1 Study material

The present study was based on a retrospective analysis of TBI patients who received early DC in the First Affiliated Hospital of Shantou University College of Medicine from August 2015 to April 2021. Our hospital is a class A, grade III medical center, located in Shantou, Guangdong Province, China, that receives about 300 TBI patients every year. We collected samples from the hospital electronic medical records, which contain variables, including gender, age, time from onset to surgery, cause of injury (including high and low falls, car accident, and other mechanisms), preoperative Glasgow Coma Scale (GCS) score, whether suffering from hypertension or diabetes, hospitalization, number of platelets, prothrombin time (PT) and international standardization ratio (INR), activated partial thrombin time (APTT), fibrinogen (FIB) count, white blood cells (WBC) count, neutrophil percentage, neutrophil count, lymphocyte percentage, lymphocyte count (LYM) and mononuclear cell count, midline shift distance, average sagittal sinus CT value, pupil dilation (unilateral or bilateral pupil dilation or no pupil dilation) and whether death occurred within 90 days.

Inclusion criteria were patients as young as 18 years but younger than 80 years who underwent unilateral or bilateral standard DC within 24 h after injury. Exclusion criteria were patients suffering from a severe chest or abdominal injury, serious heart, lung and other life‐threatening underlying diseases, or subtentorial lesions requiring surgical treatment, or patients who were lost to follow‐up or whose admission data are largely missing (patients with admission parameter loss greater than 10%). We defined standard DC as a trauma flap with a typical question mark incision and bone window size consistent with current recommendations for middle cranial fossa diameter and decompression (Hutchinson et al., 2019).

In order to reduce bias, we collected the outcome variable (whether the patient died within 90 days) and other variables separately. Patients, who did not die within 90 days and were discharged from the hospital, were followed up by telephone, and family members were asked whether the patient died within 90 days. For the average CT values of the superior sagittal sinus, CT values were measured at three different positions of the superior sagittal sinus in the last preoperative CT of the patient, and then the average values of these three positions were taken. The remaining variables, such as blood routine examination, coagulation function examination, and emergency biochemistry examination, were obtained from the records of the electronic medical record system and preoperative examinations.

### 2.2 Statistical analysis

The samples in this study were from all TBI patients receiving DC in our hospital, and the sample size was determined after rigorous screening based on inclusion and exclusion criteria. For the few missing values in the data, we used the method of multiple imputation to make up the missing values (Sterne et al., 2009). In univariate analysis, the independent sample *T* test was used for variables conforming to a normal distribution. For continuous variables that do not conform to normal distribution, the Mann–Whitney *U* test was used for nonparametric test. The *χ* ^2^ test was used for variables without inherent order. Differences were considered statistically significant at *p* < .05. Next, multivariate logistic regression analysis was performed using statistically significant variables determined by the previous analysis. According to the analysis results, variables (*p* < .05) were selected to constitute the final model, and the Hosmer–Lemeshow goodness of fit test was conducted to evaluate the model.

We used a bootstrap method (1000 replications, of 50 patients, created by random resampling) to conduct an internal verification of the model and evaluated the overall performance of the final model by using the receiver operating characteristic curve. Calibration curves, decision‐curve analysis (DCA) curve, and clinical impact curves were drawn to further illustrate and evaluate the model. To compare the predictive efficacy of the model with GCS score, we calculated the integrated discriminant improvement and net reclassification improvement. R 4.0.3 Statistical software was used for data analysis and graph drawing.

## 3 RESULTS

**FIGURE 1:**
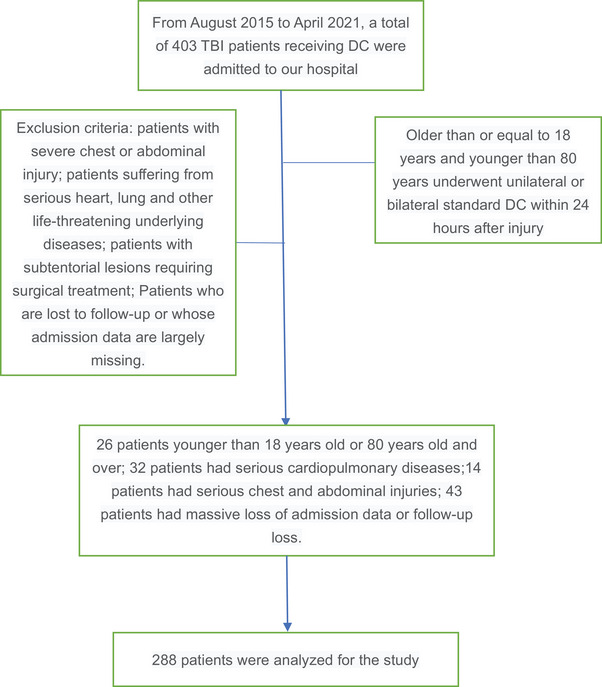
Flowchart of the study population.

From August 2015 to April 2021, a total of 403 TBI patients were admitted to our hospital and treated with DC, including 26 patients younger than 18 years old or more than 80 years old, 32 patients had serious cardiopulmonary diseases, 14 patients had serious chest and abdominal injuries, and 43 patients had a massive loss of admission data or follow‐up loss. After screening, the remaining 288 patients were used as samples for statistical analysis (Figure 1). Demographic characteristics and predictive variables of the study cohort are shown in Table 1. *p*‐Values are also provided based on univariate analysis to indicate variables that may have statistical differences. Of these, 123 patients died within 90 days. Patients with older age, shorter onset time, higher blood glucose, lower GCS score, lower platelet count (PLT), longer PT, larger INR, longer APTT, lower FIB count, lower neutrophil percentage, higher LYM percentage, higher CT value of the superior sagittal sinus, or bilateral pupil dilation were more likely to die (all *p*‐values were less than .05). There was no significant correlation between WBC count, neutrophil count, monocyte count, midline shift distance, gender, or injury mechanism and death within 90 days.

**TABLE 1:** Baseline characteristics of 288 traumatic brain injury (TBI) patients receiving decompressive craniectomy (DC) in the study cohort.

	Survived	Deceased	
Variables	*n* = 165	*n* = 123	*p*‐Value
**Age (median [IQR])**	**46.00 [31.00, 59.00]**	**53.00 [42.50, 64.50]**	**.001**
**Preoperative time, h (median [IQR])**	**6.00 [4.50, 9.00]**	**5.00 [4.00, 6.50]**	**<.001**
**GCS (median [IQR])**	**6.00 [5.00, 8.00]**	**4.00 [3.00, 5.00]**	**<.001**
**Blood glucose, mmol/L. (median [IQR])**	**10.00 [8.50, 12.10]**	**11.20 [9.15, 14.46]**	**.003**
**Osteocyte, 10^9/L (median [IQR])**	**226 [188, 269]**	**212 [173.5, 247.5]**	**.033**
**PT, s (median [IQR])**	**11.30 [10.70, 12.20]**	**12.30 [11.50, 13.20]**	**<.001**
**INR (median [IQR])**	**0.99 [0.93, 1.06]**	**1.07 [1.00, 1.15]**	**<.001**
**APTT, s (median [IQR])**	**24.00 [22.00, 27.00]**	**27.00 [24.35, 32.35]**	**<.001**
**FIB, g/L (median [IQR])**	**1.90 [1.54, 2.35]**	**1.66 [1.17, 2.20]**	**.001**
**WBC, 10^9 (median [IQR])**	**19.07 [14.36, 24.22]**	**18.84 [14.53, 24.19]**	**.828**
**Neutrophils, % (median [IQR])**	**83.35 [74.90, 87.90]**	**81.80 [63.95, 86.75]**	**.027**
**Neutrophils, 10^9/L (median [IQR])**	**16.13 [10.40, 21.26]**	**14.64 [9.84, 20.50]**	**.348**
**LYM, % (median [IQR])**	**10.60 [6.60, 18.00]**	**12.00 [7.85, 30.30]**	**.011**
**Monocytes, 10^9 (median [IQR])**	**0.89 [0.63, 1.23]**	**0.90 [0.70, 1.27]**	**.354**
**Midline shift, mm (median [IQR])**	**9.30 [6.00, 12.30]**	**10.50 [6.80, 14.85]**	**.121**
**Mean CT value, HU (median [IQR])**	**49.50 [45.50, 53.50]**	**53.00 [47.50, 56.25]**	**<.001**
**Gender = male (%)**	**128 (77.6)**	**98 (79.7)**	**.777**
**Mechanism**			**.197**
**Car accident (%)**	**104 (63.0)**	**80 (65.0)**	
**High fall injury (%)**	**19 (11.5)**	**22 (17.9)**	
**Fall damage (%)**	**31 (18.8)**	**17 (13.8)**	
**Other**	**11 (6.7)**	**4 (3.3)**	
**Hypertension = yes (%)**	**18 (10.9)**	**11 (8.9)**	**.726**
**Diabetes = yes (%)**	**7 (4.2)**	**3 (2.4)**	**.616**
**Pupil diffusion (%)**			**<.001**
**0**	**66 (40.0)**	**18 (14.6)**	
**1**	**69 (41.8)**	**33 (26.8)**	
**2**	**30 (18.2)**	**72 (58.5)**	

*Note*: *p*‐Values were calculated by the *χ* ^2^‐test (categorical variables) and Mann–Whitney *U* test (continuous variables). Statistical significance at *p* < .05.

Abbreviations: APTT, activated partial thrombin time; FIB, fibrinogen; GCS, Glasgow Coma score; INR, international standardization ratio; LYM, lymphocytes; PT, prothrombin time; WBC, white blood cell counts.

**FIGURE 2:**
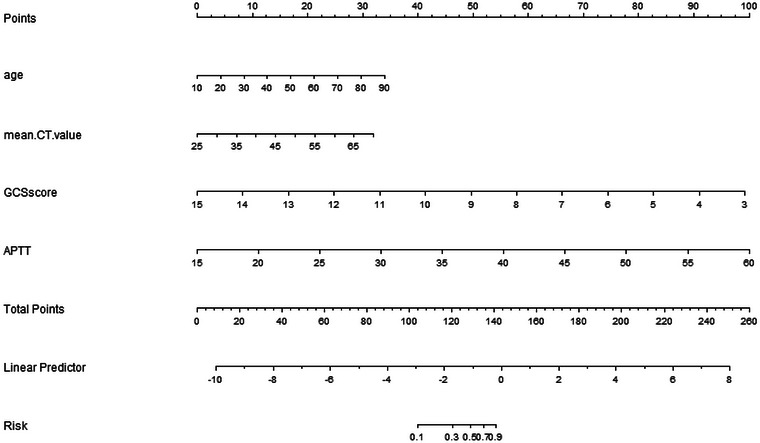
A nomogram to predict death in traumatic brain injury (TBI) patients within 30 days of receiving decompressive craniectomy (DC). In order to calculate the patient's probability of death, the points of each variable are assigned by the corresponding value on the “points” axis, and the points of each variable are plotted on the “total points” axis. The sum of all points is the patient's probability of death.

According to the outcome of univariate analysis, multivariate logistic regression analysis was performed on statistically significant predictive variables. Age (*p* = .002), APTT (*p* < .001), GCS score (*p* < .001), and mean superior sagittal sinus CT value (*p* = .039) were independent predictors of death within 90 days in TBI patients receiving DC (Table 2). Finally, age, APTT, GCS score, and mean superior sagittal sinus CT value were used as predictors to construct a prognostic model to predict the risk of death within 90 days in TBI patients receiving DC. In order to better display the model, we also constructed a nomogram (Figure 2) and calculated the death risk by using the APTT value, superior sagittal sinus CT value, age, and GCS score of TBI patients at admission in the above formula to calculate the 3‐month death risk of the patient. The equation of each variable as follows: point1 = 0.423854317 × age − 4.238543166; point2 = −8.269049991 × GCSscore + 124.03574986; point3 = 2.222222222 × APTT − 33.333333333; points = point1 + point2 + point3; lp = 0.074354216 × points − 10.665749645; Risk of death = −5.401e − 06 × points^3 + 0.002324124 × points^2 − 0.315132121 × points + 13.822596907.

**TABLE 2:** Multivariate logistic regression analysis results of the study cohort.

Variable	Odds ratio	95 %CI	*p*‐Value
Age	1.033	(1.012,1.056)	.002
Preoperative time	.954	(.885, 1.013)	.182
Mean CT value	1.056	(1.003,1.114)	.039
GCS	.624	(.487,.778)	<.001
Blood glucose	1.045	(.977,1.096)	.099
Osteocyte	.997	(.992,1.002)	.241
PT	12.048	(1.345,4421.72)	.228
INR	1.71e‐12	(1.91e − 41, 2.89e − 6)	.246
APTT	1.162	(1.068,1.271)	<.001
FIB	.957	(.835,1.236)	.564
Neutrophils	1.051	(.966,1.218)	.357
LYM	1.072	(.978,1.256)	.236
Pupil diffusion1	.905	(.382,2.1467)	.82
Pupil diffusion2	1.897	(.707,5.139)	.203

*Note*: CI indicates confidence interval.

**FIGURE 3:**
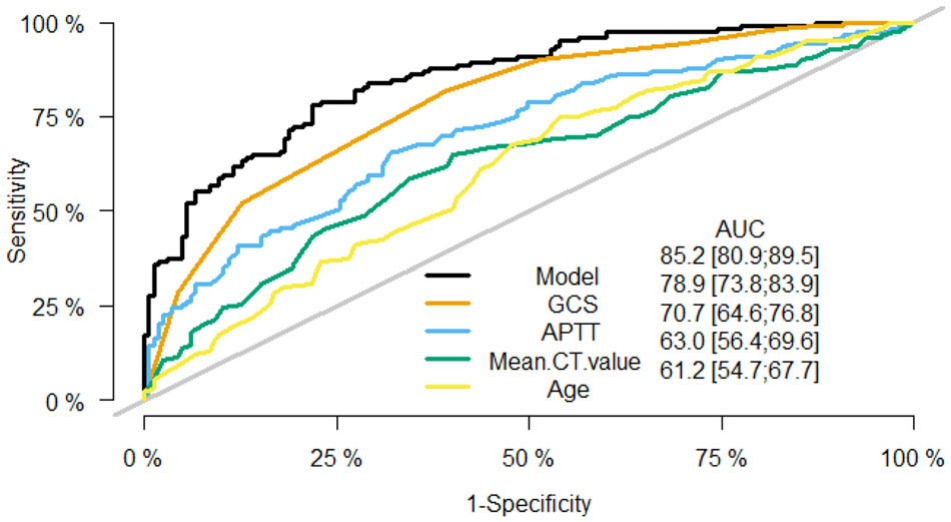
Receiver operating characteristic (ROC) curve for the model and each variable. Percentage AUC values for the model and each variable are also provided.

**FIGURE 4:**
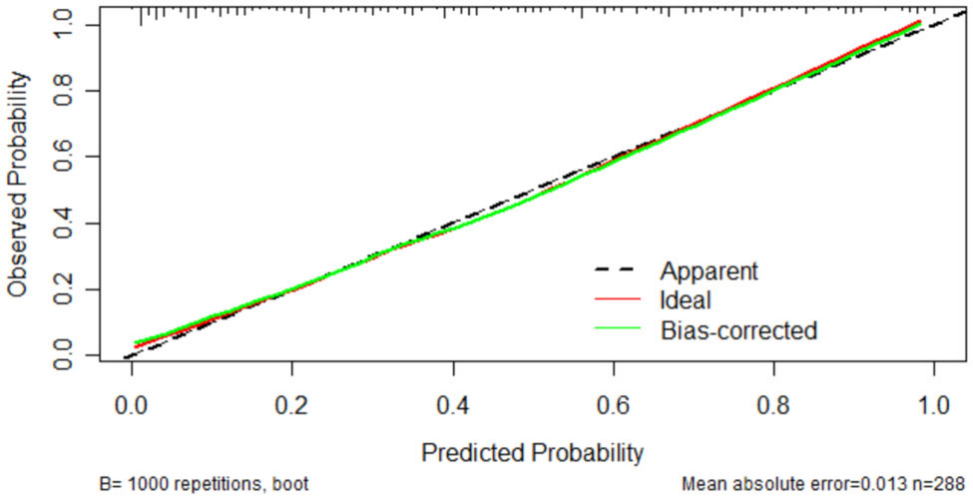
The calibration curve of the model was obtained by 1000 bootstrap resamplings with a mean absolute error rate of 0.013.

**FIGURE 5:**
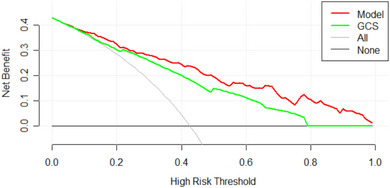
The model and Glasgow Coma Scale (GCS) prediction of patient death decision curve analysis. The net benefit curves of both were given. Red line = net benefit of treatment for patients under the guidance of this model; Green line = net benefit for patients treated under GCS guidance; Black line = net benefit of treating all patients as if no death had occurred; Light gray line = net benefit of treating all patients as if they were dead. Obviously, this model has a larger area under the net benefit curve and a higher net benefit.

Hosmer–Lemeshow goodness of fit test indicates that the model has good fit (*χ* ^2^ = 3.5403, df = 8, *p*‐value = .896). As shown in Figure 3, the area under the receiver operating characteristic curve of this model (AUC) = .852 (95% confidence interval, .8086–.8955). The prediction efficiency of this model is better than that of GCS score (AUC = .789, 95% confidence interval, .738–.839), APTT (AUC = .707, 95% confidence interval, .646–.768), mean CT value of the superior sagittal sinus (AUC = .63, 95% confidence interval, .564–.696), and age (AUC = .612, 95% confidence interval, .547–.677) and also has good predictive performance. Internal verification by bootstrap resampling gave an area under the receiver operating characteristic curve of .843. The calibration curve shows high agreement between the theoretical and actual values predicted by the model (Figure 4). To further evaluate the model, we conducted clinical impact curve analysis and clinical DCA, which showed that, compared with the GCS score, the net benefit of the model was higher in the threshold probability of 20%–100%, with good clinical predictive value (Figure 5). At the same time, we calculated the net reclassification index and the comprehensive discriminant improvement index. Compared with the GCS score, the continuous net reclassification index of the model improved by 0.6906 (95% confidence interval, .4712–.91, *p* < .001), the comprehensive discriminant improvement index increased by 0.1257 (95% confidence interval, .0852–.1662, *p* < .001).

## 4 DISCUSSION

DC is commonly used to reduce ICP in patients with TBI to improve survival and functional prognosis (Fleminger & Ponsford, 2005; Zink, 2001). However, not all patients with TBI can benefit from this procedure. Hutchinson et al. (2016) in the randomized, multicenter RESCUEicp study showed that secondary DC increased survival in patients with TBI and refractory intracranial hypertension, but it increased the proportion of persistent vegetative state and severe disability in the survival group. Furthermore, because of changes in extracranial and ICP, cerebrospinal fluid circulation disorders or abnormal coagulation, DC may lead to life‐threatening complications, such as contralateral subdural or extradural blood, enlargement of intracerebral hematoma, aggravation of cerebral contusion, hydrocephalus, and subdural spinal fluid (Aarabi et al., 2009; Carnevale et al., 2018; Di et al., 2018; Fattahian et al., 2018; Flint et al., 2008; Joseph et al., 2014; Kim et al., 2018; Nasi et al., 2018). Thus, the outcome of DC in TBI cases remains controversial. Age (Hanko et al., 2021; Huang et al., 2013; Tang et al., 2020), bilateral unreactive pupils (Tang et al., 2020), subdural hemorrhage (Chen et al., 2011; Vilcinis et al., 2017), preoperative APTT (Tang et al., 2020), and GCS scores (Collaborators et al., 2008; Hanko et al., 2021) were found to be the predicted factors for early death in TBI patients following DC. In our study, univariate and multivariate analyses showed that age, GCS score, and APTT were independent risk factors for a death of TBI patients within 3 months after receiving DC, which is similar to the findings of several previous studies. Interestingly, we also found superior sagittal sinus CT values to be an independent risk factor that few studies have addressed.

According to ROC curve analysis of all variables in this study (Figure 3), GCS score was the strongest predictor of death within 3 months of TBI patients receiving DC. The GCS is a 3‐component clinical scoring system created in 1974 by Teasdale and Jennett to continuously to assess the depth and duration of impairment of consciousness in a coma (Teasdale & Jennett, 1974). It soon became widely used to assess the severity of TBI patients, guide treatment options, and predict patient outcomes (Teasdale & Jennett, 1974). GCS score reflects the severity of TBI patients to a certain extent, which is the reason why TBI patients receiving DC with lower GCS score are more likely to die.

The APTT at admission also has good predictive efficacy in this study (AUC = .707). It is well known that PLT, PT, APTT, INR, and FIB are all parameters used to measure the coagulation of patients, and coagulation dysfunction is usually defined as one or more coagulation parameter abnormalities (Nardi et al., 2015). In univariate analysis, all were high‐risk factors. However, after multivariate analysis, only APTT was an independent predictor, and the risk of other coagulation parameters was no longer significant. This result is similar to that reported by Tang et al. (2020), except that they defined the outcome variable as the 30‐day risk of death in TBI patients treated with DC. Nakae et al. (2016) mentioned in their study that the coagulation pathway is activated in trauma patients, resulting in endogenous coagulation factors in plasma being gradually consumed, and coagulation gradually becoming abnormal, resulting in prolonged APTT. In addition, bleeding during surgery further consumes coagulation factors, aggravating coagulation dysfunction, leading to intraoperative hemostatic difficulties or postoperative brain hematoma enlargement, aggravation of brain contusion and cerebral infarction, and other fatal complications.

Age has been recognized as one of the most important prognostic factors for TBI patients receiving DC (Moskowitz et al., 2018; Zhou et al., 2019). This is consistent with our study. Univariate analysis and multivariate logistic regression analysis showed that age is an independent risk factor for death in TBI patients treated with DC, and older patients were more likely to die within 90 days. Of course, this is because with increasing age, the patient's accompanying basic diseases gradually increase, and the functional reserve of vital organs gradually decreases. Thus, DC is also a blow to older patients.

In our study, we unexpectedly found that increased mean CT value of the superior sagittal sinus is an independent risk factor for death of TBI patients requiring DC. Studies have shown that the normal range of CT values of the superior sagittal sinus is between 35HU and 65HU, and its increase often suggests the possibility of venous sinus thrombosis. Fanous et al. (2010) proposed that CT values of the superior sagittal sinus venous sinus were significantly correlated with hemoglobin content, suggesting that CT values of the superior sagittal sinus venous sinus are closely correlated with blood concentration. Erythrocyte deformability worsens and whole blood viscosity increases under hypoperfusion conditions in the porcine cerebrum (Nemeth et al., 2006). In the rat model of diffuse axonal injury, hematocrit, erythrocyte aggregation index, and whole blood viscosity gradually peak at 24 h after brain injury and decrease at 72 h (Zhou et al., 2001). Moreover, in the acute stage of TBI, calcium ions flow into cells, resulting in cytotoxic brain edema after free radical formation (Dixon et al., 1991; Maxwell et al., 1993). In the acute phase of TBI patients, upper sagittal sinus compression gradually occurs after cerebral edema, blood flow velocity slows down, and blood concentration increases, resulting in an increased CT value on non‐enhanced CT. Therefore, we suspect that the superior sagittal sinus CT value reflects the degree of cerebral edema to some extent, which may be the reason why the mean superior sagittal sinus CT value is an independent risk factor for predicting death in TBI patients requiring DC.

The model in this study was established based on clinical cases in the First Affiliated Hospital of Shantou University Medical School and has a clinically useful predictive value of 0.852 area under the curve‐receiver‐operation characteristic curve. The bootstrap resampling method was used for internal validation and gave a clinically useful predictive value was 0.843 area under the curve‐receiver‐operation characteristic curve. The calibration curve showed that the theoretical value predicted by the model is highly consistent with the actual value. The continuous net reclassification index of the model improved by 0.6906 and the comprehensive discriminant improvement index increased by 0.1257 compared with the commonly used GCS score. These data suggest that the predictive modeling of 3‐month mortality in TBI patients receiving DC, using preoperative indicators, is feasible. In addition, we also carried out clinical DCA and showed that the model has good net benefit. The aim of our study was to establish a model using only preoperative indicators to predict the 3‐month risk of death in TBI patients receiving DC. Undoubtedly, this can better guide our clinical work, provide a reference for future TBI treatment guidelines, and reduce the incidence of spending a large amount of money but failing to save patients. Compared with the random forest prediction model established by Hanko et al. (1), our model can predict the death risk of patients 3 months later only by using preoperative indicators, and the sample size in this study is larger. Therefore, the model in this study can calculate the risk of death after receiving DC in TBI patients at admission more quickly and accurately. More encouragingly, this study also explores the mean superior sagittal sinus CT value as a new predictor, which provides more consideration for TBI treatment. However, the mechanism of this indicator affecting patient prognosis still needs further research. In addition, the sample size of this study is still low, and the model has not been externally verified by cases from different hospitals, which still needs to be verified by a multicenter cohort.

## 5 CONCLUSIONS

TBI patients still have a high mortality rate after DC surgery, and the families of patients who die not only bear large costs but also spend a large amount of energy to take care of the patients. Therefore, it is important to calculate the risk of death after receiving DC in TBI patients at admission. Our study found that in addition to GCS score, age, and APTT as independent risk factors for death within 3 months after DC surgery in TBI patients, the superior sagittal sinus CT value was also independent risk factors for death within 3 months after DC surgery in TBI patients. This allowed us to build a simple mortality prediction model that includes parameters obtained at the time of patient admission and does not require patient parameters postoperatively. Although the model has shown good recognition in our patient population, external validation is still needed.

## AUTHOR CONTRIBUTIONS

**Birui Wu**: Conceptualization; formal analysis; project administration; software; writing—original draft; writing—review and editing. **Juntao Zhang**: Data curation; formal analysis. **Junchen Chen**: Data curation; formal analysis. **Xibo Sun**: Writing—review and editing. **Dianhui Tan**: Conceptualization; formal analysis; project administration; supervision; validation; writing—review and editing.

## INFORMED CONSENT

For this type of study, formal consent is not required.

### PEER REVIEW

The peer review history for this article is available at https://publons.com/publon/10.1002/brb3.3492.

## Data Availability

The data that support the findings of this study are available on request from the corresponding author [Dianhui Tan], upon reasonable request.
